# Dying as an issue of public concern: cultural scripts on palliative care in Sweden

**DOI:** 10.1007/s11019-021-10022-7

**Published:** 2021-05-06

**Authors:** Axel Agren, Ann-Charlotte Nedlund, Elisabet Cedersund, Barbro Krevers

**Affiliations:** 1 Department of Health, Medicine and Caring Sciences (HMV), Linköping University, Linköping, Sweden; 2 Department of Culture and Society (IKOS), Linköping University, Campus Norrköping, Sweden

**Keywords:** Palliative care, Cultural scripts, Policymaking, Qualitative interviews

## Abstract

In Sweden, palliative care has, over the past decades, been object to policies and guidelines with focus on how to achieve “good palliative care”. The aim of this study has been to analyse how experts make sense of the development and the current state of palliative care. Departing from this aim, focus has been on identifying how personal experiences of ‘the self’ are intertwined with culturally available meta-level concepts and how experts contribute to construct new scripts on palliative care. Twelve qualitative interviews were conducted. Four scripts were identified after analysing the empirical material: 1. *script of paths towards working within palliative care;* 2. *script of desirable and deterrent reference points*; 3. *script of tensions between improvement and bureaucracy;* and 4. *script of low status and uncertain definitions.* The findings of this study illustrate how experts in complex ways intertwine experiences of ‘the self’ with meta-levels concepts in order to make sense of the field of palliative care. The participants did not endorse one “right way” of “good” deaths. Instead, palliative care was considered to be located in a complex state where the historical development, consisting of both desirable ideals, death denials and lack of guidelines, and more recent developments of strives towards universal concepts, “improvement” and increased bureaucracy altogether played a significant role for how palliative care has developed and is organised and conducted today.

## Introduction

Policies have increasingly endorsed efficiency in order to reduce costs and improve the quality of, and access to, healthcare (Komulainen et al. 2019; Nedlund and Garpenby 2014). The prominence given to the concept of evidence-based medicine (Greenhalgh and Russell 2009) has been noted also within palliative care (Aoun and Nekolaichuk 2014) and claims have been made for palliative care to be increasingly evidence-based (Higginson 1999). The Liverpool Care Pathway (LCP) is one example of policies which have declared the need of incorporating knowledge from specialist palliative care within mainstream healthcare. The LCP was a so-called Integrated Care Pathway and was established in England and Wales during the late 1990s. The purpose of Integrated Care Pathways is to organise care, using multi-disciplinary teams for specific types of needs and patient groups, departing from evidence-based knowledge and defined steps to be followed. Following the critical public debate and the results of a review, it was abolished in 2014 (Seymour and Clark 2018). In Sweden, palliative care has, over the past decades, increasingly become an issue of public concern through policies and guidelines (Lind et al. 2017), formulated by government agencies, with focus of how to improve care, achieve universality, promote systematic use of common concepts, and develop indicators for ‘good palliative care’ (National Board of Health and Welfare 2013).

We depart from a social constructionistic perspective on organisational change (Komulainen et al. 2019), as focus is on how experts, within palliative care, make sense of the development and current state of palliative care in Sweden through linguistic representations. Attention is, in line with the overall orientation of discursive psychology, on how individuals use available linguistic resources to shape and negotiate representations of the world. Furthermore, focus is on understanding how selves, emotions and thoughts are crafted and altered through situated language use (Jørgensen and Phillips 2002: 5; Wetherell and Potter 1992). More specifically, we focus on how experts construct their views in terms of cultural scripts of dying, which can be summarised as meta-level concepts on dying available in certain social and cultural contexts (Long 2004; Seale 1998; Hunt 1992). According to Seale (1998: 68) scripts that are accessible to an individual are conditioned by social structures and the position of the individual within a society. Scripts may be used in a variety of situations and by lay persons, experts, and the media, among others (Seale 1998). In this study we will view the constructions of scripts as a part of ‘self-narratives’ (Giddens 1991:5) where individuals intertwine experiences of the ‘self’ with meta-levels concepts on palliative care.

Expert perspectives on death and dying may contribute to construct ‘normal’ or ‘good deaths’, and further, affecting those facing death to talk about feelings and experiences in certain ways (Breaden 2003). It is argued that ‘expert scripts’, i.e. commonly professionals in powerful positions, have intentions of proclaiming the ‘right way’ to die (Long 2004). However, it is noted that the paramount task of palliative care to relieve psychological, social and spiritual suffering, defined as ‘total pain’ by founder Cicely Saunders, results in professionals to be in the complex boarder between terminal sedation and causing taboos on suffering among dying persons if initial efforts to reduce suffering were not effective (Streeck 2020). Hartogh (2017) argue that a functional approach to palliative care rests on normative ideals of dying well, where physical symptoms and suffering are under medical control. According to Hartogh, the overall aim should not always be to alleviate suffering as this may not be in interest of the patient as wills to round of one life may entail suffering as a central part of dying well. The concept of ‘total pain’, was a key concept of the hospice-movement and still is guiding for palliative care today (Streeck 2020). It is however claimed, by Clark (1999), that intentions to take into all aspects of psychical suffering by acknowledging mental, spiritual and social problems also consists of disciplinary components, as these efforts entails a search for troubles in the individual’s social networks, psyche and in the soul itself. A similar argument is found regarding acceptance of dying which, according to Zimmermann (2012) who have analysed the concept of ‘acceptance’ in palliative care literature, is a requirement for effective palliative care and an aim which both families and staff strives towards and a facilitator of care. Patients who accept dying were, in comparison with those who denied dying, easier to care for and are favoured since this attitude is in line with how institutional practices within palliative care are structured. Thus, Zimmermann (2012), contend that the goal of achieving acceptance among patients illustrates elements of disciplinary power present within palliative care literature. Medical logics and neoliberal organisation of palliative care are premiered through control of medicines and symptoms and nurses’ humanistic ideals are found to be limited (Glasdam et al. 2020). It is therefore of interest to study how persons in positions of defining palliative care in Sweden describe this context. Focus on Sweden is motivated by the rapid increase of knowledge governance within palliative care over the past years. Meanwhile, studies focusing on policies within palliative care in Sweden are scarce.

The aim of this study is to analyse how experts make sense of the development and the current state of palliative care. Departing from this aim, focus is on identifying how personal experiences of ‘the self’ are intertwined with culturally available meta-level concepts and how experts contribute to construct new scripts on palliative care.

## Methods

### Design

This paper is based on an abductive qualitative interview-study with experts (Meuser and Nagel 2009), who are, or have been, active in policymaking within palliative care in Sweden.

### Participants

Twelve participants were interviewed. The participants came from different organisational contexts in Sweden; we considered it important to highlight experiences of palliative care from different settings, and the role of policies in these varying contexts. Seven were physicians and five were nurses. The participants had varying professional background and experiences. To large risk of identifying the participants. Four had a background as researchers. Five of the participants were actively involved in research, development, and education. All except one conducted clinical work to varying extents. All names of the study participants are fictional in order to deidentify them. We stopped the recruitment when we considered that the collected interview data offered a credible level of information and sufficiently contributed to ‘conceptual depth’ in relation to the aim and theoretical focus of the study (Saunders et al. 2018; Nelson 2017; Guba 1981).

### Recruitment

Participants were recruited through a process where we first identified organisations and government agencies that are active in policymaking within palliative care. In the next step, persons listed on the websites for these organisations and agencies were contacted. These persons were asked to participate in interviews for this study. When contacting them, we asked for the names of other potential study participants, which was provided on some occasions. There is a risk of bias in this process of snowball sampling, as the researcher relies on the persons who suggests potential study participants (Noy 2008). In this study, we were able to identify the suggested participants as all persons were listed on organisations and government agencies websites, and thus we could evaluate if their professional background was of relevance for the study. Furthermore, we had personal contact with all potential participants prior to the interviews, where the participants were able to decide whether the aim of the study was in line with their experiences and professional background. In most cases, however, we identified and contacted potential participants without involvement of other persons.

Participants’ informed consent was obtained when they accepted the conditions for participation, which were described in an information letter. Conditions for participation in the study was that participants had the right to leave the study without giving a reason, that all collected material would be treated confidentially, and that the participants felt that they had been sufficiently informed about the study’s purpose and the conditions for participation in order to decide on whether to participate in study or not. These conditions were based on the Swedish research ethics.^1^ The study obtained approval from the Regional Ethical Board Linkoping (Dnr 2018/293-31).

### Data collection

Prior to conducting the interviews, a semi-structured interview guide was developed to enable study participants to describe their perspectives on the development of palliative care and policymaking within this field in Sweden. As the study participants constituted a diverse group, the interview guide was flexible to reflect the professional backgrounds as well as the organisational contexts. Moreover, how the interviews proceeded was also flexible; the person conducting the interview was sensitive to how much emphasis the participants placed on each question, and the orientation of the interview was directed towards issues that the participant was seemingly most interested in and had the most insights about. Although being flexible, the interview guide consisted of certain main topics. The interviews started with the question; *tell me about yourself, what you work with and your professional background.* Departing from these questions, the participants in most interviews provided in-depth descriptions of motives for why they began to work within palliative care, their experiences of working within this context and went on to describe the development of palliative care in Sweden over the past decades. Following this stage of the interviews, questions focused on the participants involvement in policymaking within palliative care in Sweden and their experiences of these efforts and their perspectives on the role of policies in this context. The next and often last question revolved around what challenges that the participants considered to exist within palliative care today.

The context that was in focus varied during the interviews depending on the context wherein the participants were active. Each interview lasted between 40–80 min and were gathered by two of the authors (A1 and A2). The interviews were audio-recorded and transcribed word by word. Excerpts in this article were translated from Swedish into English by the authors.

### Data analysis

Throughout the process of analysis, we were inspired by Ritchie and Lewis (2003) view on analytical hierarchy in qualitative analysis. The first phase in the analysis was to get familiar with the empirical material and we engaged in thorough reading of the transcripts several times. In this phase, which Ritchie and Lewis (2003) describe as characterised by data management, we sorted the material by labelling expressions, found in the empirical material, as belonging to initially identified several themes that were close to the language of the participants. In the next phase one author (A1) conducted a first analysis and wrote a draft on the analysis. This phase was characterised by more in-depth analysis of the synthesized themes, where key dimensions and the diversities of each theme were identified. Themes were refined and overarching phenomena occurring in the material were identified, based on the accounts made by the participants, and typologies in the way that participants describe and make sense of the phenomena, which in this study was the development and current state of palliative care. Following these phases, the analysis proceeded through the theoretical lens of cultural scripts of dying (Seale 1998; Hunt 1992; Long 2004). Using this analytical focus, it was possible to discern scripts based on the participants accounts that were characterised by intertwined individual experiences with meta-levels concepts. This process was guided by expressions such as; “when I worked at…” When scripts were on a meta-level, this was found as the participants used terms such as; “palliative care today…” and “the hospice movements…” The analysis was developed and discussed by all authors (A1–A4) in an iterative process several times until the authors considered the analyse to be complete.

## Results

The results will present the four scripts found after analysing the empirical material: *script of paths towards working within palliative care; script of desirable and deterrent reference points*; *script of tensions between improvement and bureaucracy;* and *script of low status and uncertain definitions.*

### Script of paths towards working within palliative care

The participants’ accounts on why they originally chose to work within palliative care, consisted of reminiscences of past times where they described their motives for wanting to work in palliative care. Lias, who has been involved in research and policymaking over a couple of decades, viewed work within palliative care and care of the most severely ill as a calling. Excerpt 1:

*Yes, I felt a call very early on *[…]* to palliative care and to the most seriously ill, so I have always wanted that. And even when I started my specialist training, everyone knew *[…]* I was, as it were, completely dedicated from the beginning to that.*

Beatrice described her path towards palliative care as follows. Excerpt 2:

*I was from the beginning *…* an oncologist *[…]* over the years *(I became)* increasingly interested in various, what do you say, palliative issues, and working with healthcare in a different way and trying to help the rather seriously ill patients*.

For Beatrice, working in various healthcare contexts eventually led her to work within palliative care, and her interest in caring for the seriously ill grew over time. Jessica, a nurse by training, had over the years mainly worked with tasks of implementation within healthcare, and had been head of unit in different healthcare contexts. When she received an offer to be head of unit in palliative care, she accepted the offer as she felt, Excerpt 3:

[I] *have always had a commitment to the palliative patients.*

The accounts, made by the participants, often revolved around feelings of working within palliative care as a calling and commitment to terminally ill patients. For others, however, their professional career eventually led them to palliative care. When describing experiences from working within palliative care over time, recollections were made to times when palliative care did not exist as a defined field and working in long-term care institutions also meant working with death and dying where the focus was on working quickly. In these contexts, dying patients were seen as time-consuming and a cause of stress as one nurse could be responsible for up to 30 patients. The dying person was left to die and, according to Jessica, Excerpt 4:

…* you gave up, you could not do anything more. And we weren't good at pain relief either*…

Fredrik described his path towards palliative care as being motivated by what he considered to be a lack of knowledge on how to deal with death and dying, and a reluctance to face these issues within various healthcare contexts. This had an influence on his own abilities to face death and dying. Excerpt 5:

*I did not know what to say to the dying, and there was nothing about it in our training. So, we were all very perplexed, and the professor disappeared when someone was going to die, and everyone ran away*…

In this script the focus was on the individual experiences through reminiscences of past times; consequently, the participants used this script to construct a self-identity (Giddens 1991: 5) of a palliative care professional. However, this script also contained historical descriptions of the lack of knowledge and the reluctance to deal with death, dying and lack of knowledge on pain relief. Thus, although the focus was on their personal experiences, this script also reflected how end-of-life care has been provided over the past decades, and how death and dying was dealt with in healthcare and long-term care where dying persons were perceived to be time-consuming and were left to die. Thus, the culturally available ‘history’ of palliative care in Sweden were represented through ‘self-narratives’ (Giddens 1991: 244).

### Script of desirable and deterrent reference points

Throughout the interviews, the participants raised issues concerning what palliative care stands for today and used the history of palliative care as reference points. Within this script, the perspective was broadened, from a sole focus on Sweden, to discussing the international development of end-of-life care. This script was twofold, consisting of both desirable and deterrent reference points, as we will highlight in this segment.

When verbally representing the development of palliative care through these historical scripts, several participants raised the question of what will become of this field when ideals of the original social movement become accepted and institutionalised in mainstream medicine and healthcare. Most of the participants expressed the importance of upholding the original philosophies, in which viewing the dying person from a holistic approach was seen to be key. According to Lennart and Fredrik, there has been, and still is, the risk of palliative care professionals becoming merely ‘symptomatologists’ with the emphasis on symptoms being treatable with medication and they do not see the dying person. In contrast, several participants emphasised that the ability to reduce symptoms and pain for the dying person was the key component of palliative care today. Beatrice uttered the following remarks regarding these issues. Excerpt 6:

*The hospice movement in the UK, and Cicely Saunders, and everyone in the 50s and 60s, is still the ideology on which we rest. And the question is, really, how relevant is it nowadays as it is shaped by the society of the time and the care of the time. And now that has changed quite a bit*…* And now we talk more about issues of earlier identification *…* palliative care being integrated with other care*.

Throughout the interviews, the hospice movement’s care philosophy was still considered to be an important characteristic of palliative care today, but difficult to achieve in the current healthcare system. According to Lennart, discussions on the role of a holistic approach in palliative care has been ongoing for decades and is Excerpt 7:

…*extremely important, not least in today’s healthcare system where everything… becomes more and more mechanical*.

Thus, the critique of the state of healthcare and palliative care in Sweden, characterised by a primary focus on symptoms and by being more mechanical, was amplified and given meaning by contrasting the current state with the philosophies of the hospice care movement, with its emphasis on holistic care, as desirable.

The other part of this script consisted of more recent events where the rise and fall of the *Liverpool Care Pathway (LCP)* served as a reference point. This reference point was used as an example of how policies may have good intentions but could cause harm if not implemented adequately due to lack of management, insufficient introduction for staff, and lack of knowledge as highlighted by Eva Excerpt 8:

*And then you work this out in Liverpool and then you spread it all over the world *…* And from the beginning this worked very well then *…* we took it to Sweden and spread it*…* But with all these policies or guidelines or care plans there must be a proper management, there must be a purpose for this *…* And in England *…* this derailed. A decision you make at the end of life is how much fluid and nutrition does this person need when dying, should they have an intravenous drip or not?. *[…]* But then they misinterpreted this so that they said ‘When you are dying you should not have food and drink’*.

The *LCP* had, according to the participants, immense success and influence on policy development within palliative care internationally and in Sweden. The *LCP* had an impact on everyday work for most of the participants, and in particular for one participant, who taught the use of *LCP* in different contexts. However, following the overwhelming criticism and reports that led to the discontinuation of *LCP* in England and Wales, it was also aborted in Sweden. A problematic issue with the *LCP*, the participants argued, was that it became attractive for many hospitals in Sweden because following it resulted in economic compensation from the government. This, in turn, resulted in ‘the core’ (Fredrik’s expression) of palliative care being forgotten and, according to Alice, a lack of anchoring, and insufficient information and education of staff, which meant there were deficiencies in how to organise and conduct palliative care. Nevertheless, it is noteworthy that when reflecting on the *LCP* events, none of the participants were expressly critical towards its fundamental principles. It was rather the misinterpretation of its policies that was problematic, and the ‘best parts’ of *LCP* are still integrated in care plans in Sweden, according to Alice. Consequently, *LCP* served both as a deterrent example of how policies can spread rapidly while at the same time not succeeding whereas policies stemming from *LCP* had, and still have, an influence on how care plans and associated education are developed in Sweden.

‘Scandals’ of these types have however another potential. Fredrik, referred to a Swedish ‘scandal’ in 2005 where a three-year-old died. The responsible physicians claimed, nevertheless, to have provided sufficient palliative care. For Fredrik, palliative medicine has always been located in a complex tension between focus on caring for the dying person or, if there is lack of knowledge and competence, euthanasia. According to Fredrik, this tension emerges as an issue of debates in society from time to time, often related to certain incidences which gain public attention. The ‘scandal’ led, despite tragedies and public outrage, to increased attention where experts were given an opportunity to publicly inform what palliative care and palliative medicine stands for and how it should be improved. This also led to allocation of state resources, the state assigning governmental investigations with aims of improving palliative care and intense public discussions raising awareness of what palliative care and palliative medicine is. In this script, the participants’ accounts were mainly located on a meta-level where the history of the hospice movement and the rise and fall of the *LCP* were used to make the historical development and current state of healthcare and palliative care comprehensible.

### Script of tensions between improvement and bureaucracy

Much of the participants’ accounts revolved around how to improve palliative care on the one hand and the risks of too much bureaucracy on the other. It was commonly stated that palliative care had improved over the past years, and the participants’ focus was on how they had contributed to this development, which was described by Jessica in the following way Excerpt 9:

*Because we cover the entire county, we are going to build an equal care *…* an equal range of care options *[…]* and the patients should be treated equally. So that's why we have a number of overarching policies, memos and guidelines that we have developed. *[…]* we identify a need, it may have been identified by the staff, or a unit that has come up with something, and we say ‘We should highlight it on *[a region in Sweden]* level’*.

The scenario described by Jessica displays a way of working that focusses on achieving regional consensus and strives towards equal care through writing guidelines. For Beatrice, focus was on regional development and implementation, where policy documents from the Regional Cancer Centres in Cooperation (RCC)^2^ had a significant role. Excerpt 10:

*It became a good platform for working with Regional development and creating a consensus as well as creating policy documents and Regional organisational documents. And then we could *…* work it out in regional working groups and then address head of office which resulted in some new regional funds to achieve provision of more equal care. *[…] *there were really big differences when I started working on this in 2010. With very little contact with each other [across the Region] and very local products that had grown within teams*…

The improvement referred to by several participants was mainly ascribed to increased consensus on organisation, concepts, philosophical underpinnings, how care should be conducted, and how different registers and goals could be used in order to improve palliative care; here, policy documents have played an important role.

Within this script, most participants stated that policies and practice should be founded on evidence-based knowledge. Not all participants explicitly used the term evidence-based, as expressions such as best available scientific research and, simply, knowledge were also uttered. How knowledge was defined varied, nevertheless. Jessica’s understanding of the importance of regional policies being evidence-based and based on knowledge was that: Excerpt 11:

*The main point is that it should be based on science and evidence, that is the bottom line *[…]* we should only do what there is evidence for and that is usually why we say ‘Here, a PM is needed’, because when you get together such a large organisation that *[this region]* is, then there are as many wills as there are employees*.

Alice underscored the importance of integrating evidence-based knowledge with insights from reference groups consisting of professionals, researchers, patients, and relatives of the patients. Following this stage, she and her colleagues Excerpt 12;

…*got what we could get out of the evidence and picked it up in *[in the care plan]* as far as possible.*

For Alice, integrating insights from reference groups with evidence was a means to compile transparent documents that staff would feel confident in displaying to patients. Lias also emphasised that the policy works he had been involved in were largely based on gathering evidence and integrating it in different types of policy documents. Meanwhile, he argued that although much of the writings in these documents were based on scientific knowledge and evidence, he considered the process of policymaking to be highly subjective which, however, was part of the charm.

When reflecting further on the need for consensus within palliative care, contradicting perspectives emerged. Efforts to increase consensus were considered to be achieved by formulating standards, goals, departing from evidence-based knowledge, and using forms, registers and policies. The same measures were however argued to increase the workload. Too much bureaucracy was also considered to potentially result in the loss of the original core of a holistic approach, where focus was on attending to all aspects of the dying person. Eva had this view on documentation and implementation of new policies. Excerpt 13:

*The risk is that the policy will become like the centre *[…]* You put this in the journal system and it was very practical because then you only had to put a click like this when you had done things *…* And then you were sure to click in all the boxes *[…]* if you don’t know how to use it then it will be wrong. If I take a violin and play it, it sounds awful, I cannot play the violin, but it is not the violin’s fault*…

Klas broadened the perspective, arguing that the state of the Swedish healthcare sector in general is Excerpt 14;

…*extremely steered by policies and extreme amount of systematisation, *(which have led to)* policy fatigue.*

However, he argued that this is not the case within palliative care today, as professions in palliative care are working towards the same goals. The main assessment by the study participants was, nevertheless, that there is too much focus on documentation and bureaucracy. Lias argued that the entire healthcare sector and palliative care in Sweden is under the burden of documentation which takes away the joy, and is Excerpt 15;

…*opposite of the idea of palliative care which is really about seeing the whole person from all perspectives including the existential*.

When describing the needs for achieving, mainly, regional consensus and working with evidence-based knowledge, focus was on the self. When expanding the perspectives to palliative care and the healthcare sector in general, meta-level concepts were deployed to represent the problems associated with increased bureaucracy. Improvement was thus represented through self-narratives, whilst meta-level concepts were used to make sense of bureaucracy.

### Script of low status and uncertain definitions

Although it was commonly stated that palliative care had improved, palliative care was meanwhile considered to attract little attention and was alleged to have low status in society and healthcare today. The same participants who stated that palliative care had improved would later in the interviews depart from more critical perspectives where difficulties in gaining acknowledgment and reaching consensus were addressed.

The low status of palliative care in society, and in healthcare in particular, was emphasised by a majority of the study participants. One main reason for this low status, the participants argued, is that people in general are not interested in talking about death and dying. In the healthcare context, this low status was mostly rooted in the fact that healthcare services are still dominated by the aim of saving and prolonging lives. Several of the study participants expressed that they had made attempts to increase attention for palliative care within healthcare and society. Efforts that were, however, continuously met with resistance, and palliative care was acknowledged neither by the healthcare services nor by those physicians with a high degree of authority in Sweden. Some of the participants had initiated dialogues with units at hospitals on the need for palliative expertise. However, these efforts were not taken up, and it was challenging for professionals with palliative expertise to find a place in hospitals or in healthcare in general. When approaching a unit in a hospital with the intention of sharing palliative expertise, Lias got the reply Excerpt 16:

*At this hospital nobody dies, *…

despite the fact that statistics, according to Lias, demonstrated that people did in fact die at that hospital.

Endeavours to reach consensus were also obstructed, the participants argued, by the divide between specialised and general palliative care, which resulted in there being no clear definition of the content of the two types of care and there were ongoing efforts within organisations to map out what characterised specialised palliative care and how to measure its quality. According to Beatrice it was difficult to recruit representatives from general palliative care to policymaking, resulting in a lack of perspectives from this domain. Beatrice reflected on why general palliative care, in her view, is given less attention. Excerpt 17:

*I think that pretty much, of course, healthcare reflects social values and *…* values that exist in life in modern society in general*…* it becomes an expression for what we think of the weaker and the dying and so on. *[…]* you maybe think that the older patient may be able to manage his own aging and dying quite well, but not everyone does. And in acute care, illness and dying is still seen very much to be a failure and something to avoid*…

These tendencies, Beatrice argued, led to an absence of knowledge about the palliative needs of older people because ageing and dying are not prioritised issues since the general view, according to Beatrice, is that dying is a process that older persons can manage by themselves. Karin noted that, after giving lectures to general healthcare staff about palliative care, they did not view themselves as professions that are a part of general palliative care. In their view, Karin stated, palliative care was thought to be provided by specialised palliative teams. In line with this, Sofie thought that physicians in palliative medicine are skilled and often contribute to developing palliative care, but it is mainly their perspectives that dominate in policies and practice, whereas insights from assistant nurses and nurses are not taken into account to the same extent.

## Discussion

Based on the findings of this study, the identified scripts did consist of notions of what constitutes ‘good’ and ‘bad’ care of dying people. These understandings were however rather complex and consisted of ‘self-narratives’ which were intertwined with meta-level concepts on the history and current state of when dying becomes a public concern as in the case of palliative care. It is argued that expert scripts of dying often consist of claims for the right way of dying (Long 2004). Furthermore, how to enhance good care of dying is central within palliative care and professionals’ beliefs have a considerable influence on what characterises good dying (McNamara 2004; Payne et al. 1996; Semino et al. 2014).

According to the participants, palliative care had migrated from the ideals of the hospice movement with its emphasis on holistic care, and this contrasted with the present time which prioritises rational ways of organising and providing palliative care which not only emphasise routinisation, bureaucracy, technical and medical expertise, but also the entry of evidence-based medicine in the realm of palliative care. The history of the hospice movement was constructed by the participants as a desirable reference point, and the rise and fall of *LCP* as a deterrent. It would be tempting to view this as merely a division between the good death, as represented by the history of the hospice movement, and the bad institutionalised and bureaucratised death of today, associated with the *LCP.* When analysing the scripts further, the accounts were however not coherent. The common view that death and dying in postmodern societies have a component of nostalgia (Walter 1994) was only relevant in relation to the hospice movement’s international history. When reminiscing about end-of-life care in Sweden in past decades, the participants were more critical. It was through these recollections to times when end-of-life care in Sweden was insufficient that the improvement in palliative care in Sweden today was given meaning. By understanding what constituted ‘bad’ end-of-life care in the past, it was possible to construct what characterises ‘good’ palliative care today. The *LCP,* on the other hand, which is, according to Seymour and Clark (2018), characterised by a rational and standardised care with steps to follow and evidence-based guidelines, could be interpreted as representing increased bureaucracy. The participants argued, however, that the *LCP* had several positive components which could improve palliative care and the ‘best parts’ continue to form a part of national care plans in Sweden. Rather, the problem with the *LCP* was the lack of introduction for staff and the misinterpretation of its purpose.

The scripts illustrated how dying have become an issue of public concern through “scandals” within end-of-life care, increase in policies and knowledge production, formulated by government agencies in Sweden, public debates on what palliative care is and the expansion of palliative care within healthcare and medicine. Nevertheless, this field was still considered to have low status mainly because of death denial which, according to the participants, still existed in society and healthcare. Thus, these perspectives resembled that of Western societies as death denying because of medical and technical developments and relocation of the place of death and dying from the home to the hospital (Zimmermann and Rodin 2004; Tradii and Robert 2019). This, it is argued, has ultimately led to death and dying becoming taboo and only treated by medical interventions (Gorer 1955; Aries 1974). Another barrier for achieving consensus and wider recognition was of an internal character because specialised palliative care, in contrast to general palliative care, was regarded as having more prominence and often understood to be *the* palliative care. Representations of general palliative care focused on uncertainty regarding content, definitions, and professional identity such that general healthcare staff did not view themselves as working within general palliative care. Furthermore, general palliative care was argued to be unacknowledged in policies because staff in this sphere did not have the time to take part in policymaking. Meanwhile, counter scripts emerged stating that specialised palliative care also lacked established definitions of content and goals. Endeavours to understand and define what palliative care is, resemble what Hibbert et al. (2003) label a crisis of definitions, where strivings towards coherence and definitions of stable boundaries for palliative care expertise is seen as necessary in the medical world, particularly when the scope of palliative care is expanding from the original focus on cancer patients. This crisis was, according to the participants of this study, indeed present for both general and specialised palliative care but was greater for general palliative care. We would argue, however, that the crisis of definitions was not solely central within this script but permeated all scripts. The improvement of palliative care was ascribed to increased consensus on concepts. Concomitantly, palliative care was still considered to lack stable definitions, and there was a continuous need for improvement through new and clearer definitions. Because society and the healthcare sector were still, according to the participants, to some extent death-denying it was necessary to have stable definitions to bring attention to palliative care, and to increase knowledge about death and dying. Efforts to achieve consensus through fixed definitions, guidelines, registers, and policies were however part of the tension in the *script of improvement and too much bureaucracy*, as too much focus on consensus and definitions would lead to prioritising bureaucracy and medical treatment with the risk of increased workloads. Thus, the efforts to achieve improvement had simultaneously resulted in a diminishing of the core of palliative care. The ‘crisis of definitions’ found in the scripts highlight, however, how issues of where to set limits and boundaries for palliative care is a key issue in times of increased medicalisation and when palliative care is expanding from cancer to also other illnesses, and efforts to integrate palliative care into mainstream healthcare.

Altogether these scripts displayed how the participants, through constructing scripts on the development and current state of palliative care, negotiated what constitutes ‘good’ and ‘bad’ deaths and how both the past and present was considered to contain elements of ‘good’ and ‘bad’ deaths. Consequently, the participants did not endorse one ‘right way’ of how to enhance a ‘good’ death. Instead, the scripts displayed how views of dying within the healthcare sector, in society as a whole, and increased bureaucracy altogether have played a role for the development palliative care and how care at the end of life is organised and conducted.

## Data Availability

The gathered empirical material for this study is not accessible for others due to the risk of identification of the study participants.

## Footnotes

1 https://www.vr.se/download/18.2412c5311624176023d25b05/1555332112063/God-forskningssed_VR_2017.pdf18/5–20.

2 Regional Cancer Centres in Cooperation (RCC) is organised by Sweden’s municipalities and regions (SKR), which is a member and employer organisation for all municipalities and regions. RCC have been active in publishing policy-documents in palliative care.
